# Supplementary material for: Long-Term Monitoring Reveals Changes in the Small Mammal Community Composition and Co-Occurrence Patterns in the Diannan Area of Yunnan, China
Source: Biology (Basel). 2025 Jul 21;14(7):897. doi: 10.3390/biology14070897 (PMC12292743; doi:10.3390/biology14070897)
Supplement: Supplementary file 1 [file biology-14-00897-s001.zip › biology-3739985-supplementary.pdf]

TableS1. Changes in relative abundance (%) of different small mammals in Diannan, Yunnan Province, China, 2005-2017. The red text indicates the relative abundance of the dominant species in that year.

Species	Relative abundance (%)													
	2005	2006	2007	2008	2009	2010	2011	2012	2013	2014	2015	2016	2017	Average
Eothenomys miletus	35.23	31.17	42.15	7.06	36.44	36.84	21.04	34.38	35.86	35.52	52.30	39.81	45.01	33.36
Apodemus chevrier	37.25	39.11	37.50	67.55	45.85	28.69	39.33	38.86	44.93	40.43	34.99	42.27	41.09	41.32
Rattus norvegicus	10.58	12.41	8.65	9.99	8.17	14.77	13.26	8.85	6.29	7.93	3.25	4.42	4.15	9.47
Rattus flavipectus	6.38	7.07	5.29	7.42	5.40	12.22	10.82	8.29	6.23	8.10	3.87	3.56	4.42	7.37
Apodemus speciosus	3.81	1.59	0.48	4.03	1.45	2.21	6.71	3.81	2.84	4.66	2.89	1.30	1.55	3.13
Apodemus sylvaticus	1.09	1.59	0.64	-	0.13	-	1.83	1.68	0.34	0.17	-	0.33	0.05	0.68
Micromys erythrotis	1.24	0.43	0.64	2.20	2.24	3.74	1.52	0.78	1.02	1.12	0.43	0.63	1.78	1.40
Rattus nitidus	1.24	1.44	0.64	0.28	0.13	-	1.07	0.22	0.54	0.69	0.98	0.20	0.59	0.66
Dremomys pernyi	0.16	0.43	0.48	0.18	0.26	-	-	-	-	-	-	-	-	0.14
Suncus murinus	0.16	-	0.64	0.18	0.13	-	-	0.11	0.27	-	-	0.43	0.27	0.14
Mus musculus	0.93	0.14	0.32	-	0.13	-	-	-	-	-	-	1.10	-	0.14
Tupaia belangeri	1.09	0.29	1.92	1.01	0.53	1.19	2.59	1.01	1.08	0.86	0.68	0.90	0.68	1.11
Sciurotamias forresti	0.08	1.44	0.16	-	0.13	-	-	0.11	-	-	-	-	-	0.17
Sciurus iginventris	0.16	0.72	-	-	-	-	0.61	0.11	-	-	-	-	-	0.15

Species	Relative abundance (%)													
	2005	2006	2007	2008	2009	2010	2011	2012	2013	2014	2015	2016	2017	Average
Niviventer andersoni	0.08	1.01	0.32	0.09	-	-	-	0.34	-	0.09	-	0.20	-	0.17
Crocidura attenuata	-	0.29	-	-	-	0.17	0.46	0.34	0.07	0.17	0.06	1.26	0.09	0.14
Anourosorex squamipes	0.16	-	-	-	-	0.17	0.15	0.11	0.14	0.17	0.17	0.60	-	0.09
Niviventer confucianus	0.08	0.14	-	-	-	-	0.15	0.45	0.34	-	-	0.03	0.09	0.12
Rattus andamanensis	0.08	0.14	-	-	-	-	0.15	0.11	-	0.09	0.09	2.96	0.18	0.07
Mustela sibirica	0.08	0.14	-	-	-	-	-	0.45	-	-	-	-	-	0.08
Parascaptor leucura	0.08	0.29	0.16	-	-	-	0.15	-	0.07	0.07	0.07	-	0.05	0.05
Niviventer fulvescens	0.08	0.14	-	-	-	-	0.15	-	-	-	-	-	-	0.04

TableS2. The *P* - values of differences in Shannon's Index, Margalef Richness Index and Gini-Simpson Index between every two years from 2005 - 2017 in Diannan area, Yunnan province, China.The bolded parts indicate significant differences.

Years	Shannon-Wiener diversity index	Margalef richness index	Simpson dominance index	Pielou evenness index
2005/2006	0.453	0.644	0.862	0.174
2005/2007	**0.004**	**0.038**	**0.003**	0.434
2005/2008	0.073	**0.006**	0.118	0.418
2005/2009	**0.001**	**0.010**	**0.004**	0.664
2005/2010	0.285	**0.007**	0.885	**0.004**
2005/2011	0.260	0.751	0.255	**0.003**
2005/2012	0.453	0.885	0.417	0.707
2005/2013	**0.018**	**0.015**	0.071	0.488
2005/2014	0.337	0.509	0.767	0.544
2005/2015	**0.001**	0.057	**<0.001**	**0.001**
2005/2016	**0.033**	0.817	**0.001**	**0.004**

Years	Shannon-Wiener diversity index	Margalef richness index	Simpson dominance index	Pielou evenness index
2005/2017	**0.001**	**0.008**	**<0.001**	**0.004**
2006/2007	**0.024**	0.126	**0.046**	0.193
2006/2008	0.106	**0.023**	0.133	0.340
2006/2009	**0.014**	**0.033**	**0.032**	0.285
2006/2010	0.488	**0.015**	0.772	0.073
2006/2011	0.133	0.931	0.156	0.073
2006/2012	0.977	0.707	0.840	0.644
2006/2013	0.094	**0.030**	0.183	0.729
2006/2014	0.862	0.312	0.707	0.954
2006/2015	**0.005**	0.126	**0.002**	**0.001**
2006/2016	0.184	0.214	0.064	**0.002**
2006/2017	**0.012**	**0.021**	**0.012**	**0.003**
2007/2008	0.603	0.386	0.686	0.488
2007/2009	0.563	0.371	0.977	0.954
2007/2010	0.083	0.341	**0.013**	**0.005**
2007/2011	**0.001**	0.204	**0.003**	**0.009**
2007/2012	**0.018**	0.106	**0.049**	0.370
2007/2013	0.355	0.470	0.156	0.355
2007/2014	**0.038**	0.419	0.064	0.326
2007/2015	0.285	0.977	0.078	**0.015**
2007/2016	0.370	**0.012**	0.817	**0.011**
2007/2017	0.435	0.402	0.486	0.108
2008/2009	0.603	0.977	0.435	0.386
2008/2010	0.204	0.977	0.105	**0.043**
2008/2011	0.065	**0.015**	**0.043**	0.060
2008/2012	0.088	**0.013**	0.156	0.355
2008/2013	0.386	0.931	0.271	0.312
2008/2014	0.065	**0.043**	0.078	0.193
2008/2015	0.885	0.285	0.977	0.544
2008/2016	0.272	**0.001**	0.370	0.931
2008/2017	0.419	0.954	0.434	0.781
2009/2010	**0.009**	0.840	**0.013**	**0.009**
2009/2011	**0.001**	**0.033**	**0.004**	**0.022**
2009/2012	**0.007**	**0.015**	**0.030**	0.603
2009/2013	0.113	0.840	0.139	0.623
2009/2014	**0.010**	0.057	0.053	0.355
2009/2015	0.299	0.285	0.099	**0.030**
2009/2016	0.094	**0.002**	0.582	**0.012**
2009/2017	0.795	0.954	0.862	0.217
2010/2011	**0.006**	**0.017**	0.130	0.772

Years	Shannon-Wiener diversity index	Margalef richness index	Simpson dominance index	Pielou evenness index
2010/2012	0.644	**0.009**	0.582	**0.019**
2010/2013	0.175	0.954	0.117	0.053
2010/2014	0.285	**0.033**	0.664	0.083
2010/2015	**0.007**	0.157	**0.002**	**<0.001**
2010/2016	0.298	**0.001**	**0.045**	**<0.001**
2010/2017	0.057	0.795	**0.011**	**<0.001**
2011/2012	0.166	0.885	0.134	**0.033**
2011/2013	**0.002**	**0.021**	**0.008**	0.069
2011/2014	0.175	0.194	0.505	0.194
2011/2015	**0.001**	0.119	**0.001**	**<0.001**
2011/2016	**0.010**	0.507	**0.026**	**<0.001**
2011/2017	**0.001**	**0.015**	**0.006**	**<0.001**
2012/2013	0.094	**0.017**	0.368	0.966
2012/2014	0.931	0.236	0.794	0.686
2012/2015	**0.003**	**0.106**	**0.001**	**0.004**
2012/2016	0.149	0.729	0.060	**0.003**
2012/2017	**0.002**	**0.017**	0.064	**0.026**
2013/2014	0.094	**0.040**	0.147	0.644
2013/2015	**0.030**	0.532	**0.003**	**0.007**
2013/2016	0.684	**0.002**	0.773	**0.007**
2013/2017	0.343	0.885	0.331	**0.039**
2014/2015	**0.005**	0.340	**0.004**	**0.007**
2014/2016	0.258	0.098	0.213	**0.003**
2014/2017	**0.014**	0.100	0.067	0.060
2015/2016	**0.026**	**0.010**	**0.019**	0.885
2015/2017	0.214	0.441	**0.037**	0.217
2016/2017	0.068	**0.002**	0.233	0.139
